# Biological Characterization and Evolutionary Dynamics of Pigeon Paramyxovirus Type 1 in China

**DOI:** 10.3389/fvets.2021.721102

**Published:** 2021-10-13

**Authors:** Tiansong Zhan, Dongchang He, Xiaolong Lu, Tianxing Liao, Wenli Wang, Qing Chen, Xiaowen Liu, Min Gu, Xiaoquan Wang, Shunlin Hu, Xiufan Liu

**Affiliations:** ^1^Animal Infectious Disease Laboratory, School of Veterinary Medicine, Yangzhou University, Yangzhou, China; ^2^Jiangsu Co-innovation Center for Prevention and Control of Important Animal Infectious Diseases and Zoonosis, Yangzhou University, Yangzhou, China; ^3^Jiangsu Key Laboratory of Zoonosis, Yangzhou University, Yangzhou, China

**Keywords:** pigeon paramyxovirus type 1, evolutionary dynamics, Bayesian phylogenetic analysis, host preference, biological characterization

## Abstract

Pigeon paramyxovirus type 1 (PPMV-1) is considered as an antigenic variant of Newcastle disease virus (NDV) which has an obvious host preference for pigeons and has caused significant economic losses to the global poultry industry. The evolutionary dynamics of PPMV-1 in China, however, are poorly understood. In this study, we characterized seven PPMV-1 isolates from diseased pigeons collected in Jiangsu, Anhui, and Henan provinces during 2020. Phylogenetic analysis revealed that seven isolates belonged to sub-genotype VI.2.1.1.2.2. Biological characterization indicated that seven isolates were mesogenic based on the mean death time (69.6–91.2 h) and intracerebral pathogenicity index (1.19–1.40) and had similar growth kinetics in chicken embryos and CEFs. Furthermore, the four representative viruses (AH/01/20/Pi, JS/06/20/Pi, HN/01/20/Pi, and HN/02/20/Pi) could result in marked cytopathic effects (CPE) in CEFs and induced syncytium formation in Vero cells. Our Bayesian phylogenetic analysis showed that PPMV-1 might first emerge in East China in 1974 and East China had the highest genotypic diversity of PPMV-1. Besides, phylogeographic analysis indicated that East China and South China were probably the major epicenters of dissemination of PPMV-1 in China. Selection pressure analysis and amino acid substitutions analysis revealed that the viral replication complex (NP, P, and L proteins) was likely related with the host preference of PPMV-1. Collectively, this study uncovered the epidemiology and evolutionary dynamics of PPMV-1 circulating in China, emphasizing the importance of strengthening the monitoring of PPMV-1 in East China and South China and providing significant clues for further studies on the molecular mechanism underlying host preference of PPMV-1.

## Introduction

Newcastle disease (ND), caused by virulent Newcastle disease virus (NDV), is an acute and highly contagious disease that has caused significant economic losses to the global poultry industry (1, 2). NDV is classified as a member of the genus *Orthoavulavirus*, family *Paramyxoviridae* (3). The genome of NDV is ~15.2 kb in length (4) and encodes six structural proteins, including nucleocapsid (NP) protein, phosphoprotein (P), matrix (M) protein, fusion (F) protein, hemagglutinin-neuraminidase (HN) protein, and large (L) protein (5). Additionally, the P gene encodes two additional non-structural proteins (V and W) (6, 7). NDV isolates can be grouped into three pathotypes according to their degree of virulence in chickens: velogenic (highly virulent), mesogenic (moderately virulent) and lentogenic (avirulent or low virulent) (8). NDV isolates are phylogenetically categorized into two distinct classes, class I and class II (9). Class I viruses comprise a single genotype and are frequently isolated from wild birds and domestic waterfowls (10–12). Class II viruses have emerged as a major cause of ND outbreaks worldwide and can be segregated into 20 genotypes (I to XXI, no XV) (13).

Pigeon paramyxovirus type 1 (PPMV-1) is an antigenic variant of NDV which has a high host preference for pigeons (14, 15). PPMV-1 was first identified in the Middle East in the late 1970s and subsequently spread rapidly throughout Europe, resulting in the third ND pandemic during the 1980s (16–18). Currently, PPMV-1 is still prevalent in several countries worldwide (19–22). Pigeons are mainly split into three types: meat-type pigeons, homing pigeons, and fancy pigeons, with meat-type pigeons being the most common breed in China. With the rapid development of China's social economy and the continuous improvement in people's quality of life in recent years, the pigeon industry has also developed rapidly, but the epidemic situation of diseases caused by PPMV-1 is not optimistic. Since its first isolation in China in the 1980s, PPMV-1 has long been prevalent in numerous provinces and regions of China, seriously impeding the development of the pigeon industry (23–27). As a result, it is critical to strengthen the epidemiological surveillance of PPMV-1 in China. Bayesian phylodynamic inference is widely regarded as one of the most employed methods for speculating on the origin of epidemics, tracking the geographic spread and transmission dynamics of infectious pathogens (28). Several previous studies have suggested that PPMV-1 of China might have originated in Europe (14, 27, 29, 30). However, the evolutionary and transmission dynamics of PPMV-1 in China remain not well-understood.

In this study, we identified seven PPMV-1 isolates from diseased pigeons collected in Jiangsu, Anhui and Henan Provinces from January 2020 to December 2020 and determined the biological characteristics of these isolates. We further deeply investigated the prevalence, evolution and transmission dynamics of PPMV-1 prevailing in China. Moreover, we also performed the selection pressure analysis of different genes of PPMV-1 in China. Additionally, we identified specific amino acid substitutions in different proteins of PPMV-1 isolates by comparing the complete genome sequences of these viruses with those of chicken-origin NDVs. This study contributes to a more systematic and comprehensive understanding of PPMV-1 prevailing in China.

## Materials and Methods

### Cells, Eggs, and Animals

Primary chicken embryo fibroblasts (CEFs) were prepared and cultured as described previously (31). All embryonated SPF chicken eggs were provided from Zhejiang Lihua Agricultural Technology Co., Ltd. (Zhejiang, China). One-day-old chicks were hatched in our laboratory from SPF embryonated chicken eggs.

### Virus Isolation and Biological Characterization

Seven PPMV-1 strains were isolated from pigeons in Jiangsu, Anhui, and Henan Provinces in China during January-December 2020. Table 1 presents the detailed information about the virus names, hosts, isolation dates, locations, the size of the flock, the clinical outcome for the flock and the age of the pigeons. Hemagglutination (HA), hemagglutination inhibition (HI), and reverse transcription-polymerase chain reaction (RT-PCR) assays were used for detection and identification of PPMV-1 isolates. All PPMV-1 isolates were then purified three times by plaque assay on CEF cells (32) and subsequently propagated in 10-day-old SPF chicken embryos. The virulence of all PPMV-1 isolates was evaluated by mean death time (MDT) in 10-day-old SPF embryonated chicken eggs and intracerebral pathogenicity index (ICPI) in 1-day-old SPF chicks (33): the MDT test in 10-day-old SPF chicken embryos (hours) (>90, lentogenic strains; 60–90, mesogenic strains; <60, velogenic strains), and the ICPI test in 1-day-old SPF chicks (<0.7, lentogenic strains; 0.7–1.5, mesogenic strains; >1.5, velogenic strains) (34).

**Table 1:** The details of 7 PPMV isolates in this study.

**Isolates**	**Host**	**Age**	**Isolation date**	**Isolation location**	**Flock size**	**Morbidity**	**Mortality**	**HA titer (log2)**	**Genotype**	**F-protein cleavage site**	**GenBank accession no**.
AH/01/20/Pi	Homing pigeon	2 years	Jan 2020	Anhui	70	28.6%	14.3%	6	VI.2.1.1.2.2	^112^RRQKRF^117^	MW271789
JS/01/20/Pi	Homing pigeon	1 year	Jan 2020	Jiangsu	80	31.3%	18.8%	6	VI.2.1.1.2.2	^112^RRQKRF^117^	MW271790
JS/02/20/Pi	Meat-type pigeon	7 months	Jan 2020	Jiangsu	25	24%	20%	5	VI.2.1.1.2.2	^112^RRQKRF^117^	MZ223436
JS/06/20/Pi	Meat-type pigeon	2 months	Jun 2020	Jiangsu	600	4.2%	2.5%	6	VI.2.1.1.2.2	^112^RRQKRF^117^	MW271791
JS/07/20/Pi	Meat-type pigeon	3 months	Jul 2020	Jiangsu	100	/^a^	10%	5	VI.2.1.1.2.2	^112^RRQKRF^117^	MW271792
HN/01/20/Pi	Meat-type pigeon	14 days	Nov 2020	Henan	/	/	/	6	VI.2.1.1.2.2	^112^RRQKRF^117^	MZ223437
HN/02/20/Pi	Meat-type pigeon	20 days	Dec 2020	Henan	/	/	/	6	VI.2.1.1.2.2	^112^RRQKRF^117^	MZ223438

a *Not determined*.

To access the growth kinetics of the PPMV-1 isolates in chicken embryos, the allantoic cavities of 10-day-old SPF embryonated chicken eggs were inoculated with 100 TCID_50_ of each virus per embryo. Three chicken embryos were chilled and allantoic fluid containing virus was harvested at 24 and 48 h post-infection (hpi). Additionally, to determine the growth kinetics of the PPMV-1 isolates in chicken embryo fibroblasts (CEFs), primary CEFs grown in six-well plates were infected with the PPMV-1 isolates at a multiplicity of infection (MOI) of 0.01. At 24 and 48 hpi, supernatant samples were taken and replaced with an equal amount of fresh medium. Virus titers were evaluated by performing 50% tissue culture infective dose (TCID_50_) assays in CEF cells. The TCID_50_ was calculated by the Reed-Muench method.

For the cytopathic effect assay, primary CEFs were grown in six-well plates and infected with the four PPMV-1 isolates (AH/01/20/Pi, JS/06/20/Pi, HN/01/20/Pi, and HN/02/20/Pi) at an MOI of 0.01. To observe the cytopathic effect (CPE), cells were washed three times with phosphate-buffer saline (PBS), fixed with methanol and stained with Giemsa at 48 hpi.

The fusogenic abilities of the four PPMV-1 isolates (AH/01/20/Pi, JS/06/20/Pi, HN/01/20/Pi, and HN/02/20/Pi) were examined by the fusion index. Briefly, the viruses were inoculated onto Vero cells grown in six-well plates at an MOI of 0.1. At 36 hpi, cells were washed three times with PBS, fixed in ice-cooled methanol for 20 min, and stained with Giemsa for syncytia observation under an inverted microscope. The fusion index was determined as the ratio of the number of nuclei to the total number of cells and was normalized to the PPMV-1 isolate, AH/01/20/Pi, set as 100 (35, 36).

### Whole-Genome Sequencing

Viral RNA was extracted directly from the collected allantoic fluid with EasyPure® Viral DNA/RNA Kit (TransGen Biotech, Beijing, China). PCR amplification was conducted using 11 pairs of universal primers specific to the complete genomes of PPMV-1. The universal primers used in the present study are shown in Table 2. To obtain the 3′ and 5′ ends of the viral genomes, the PCR-generated fragments containing the leader and trailer sequences of all 7 PPMV-1 isolates were cloned into the pEASY®-Blunt Zero vector (TransGen, China) for sequencing. The sequencing was carried out by Tsingke Biological Technology (Nanjing, China). All assembled genomes were submitted to GenBank (see Table 1 for accession numbers).

**Table 2:** Universal Primers for the amplification of the whole genome of PPMV-1 isolates.

**Primer name**	**Forward primer (5′-3′)**	**Reverse primer (5′-3′)**	**Product size (bp)**
A	ACCAAACAGAGAATCYGTGAG	CGGGYGAATGGTGCTCTGT	1,534
B	GGTCCTYACTGGGCTCAGCRA	TGTGTCACGTAAAGGGAYGGATCTC	1,483
C	TCAAAGCAGACATCCTCCA	AAATGTMACTTTCTTTCCCCTCT	1,421
D	ATGTCACTATTGAYGTGGAGGTA	GGACAAGTGCTGAGGCAAAYC	1,593
E	GACTCACAGACTCAACTCTTGG	AGTAGAAAAGAATACCCTCCC	1,709
F	GCTGCACTCGGATACCCTCA	GCAGAYTCAAGTATTTTCTTCCATT	1,643
G	CCAGAGTCACATCTATCYTCYCCAT	AAAATCCGCCCATTAACCTT	1,616
H	AGAAGAAACAGGTRAAGGAGG	ACCRCTTCTTGATTGAGTAGRA	1,621
I	TCAAGCGATTAGAAGCARTGGGG	AGTGAATGATGGGRTGAGAAAT	1,734
J	ACACACGAAATTGGATTAGTGAAGC	TTAGACAGAAGTGTACCGTGC	1,630
K	TGACCTCRGATAAGGCAGTGGG	ACCAAACAAAGATTTRGTGAA	1,237

### Collection of Sequences

A total of 427 complete F gene sequences of NDV (PPMV-1 and chicken-origin NDV belonging to genotype XX) were collected from GenBank (as of February 2021) and were aligned by using Multiple Alignment with Fast Fourier Transformation (MAFFT v.7.4.50). Additionally, PPMV-1 isolates from China were selected and a dataset containing 186 complete F gene sequences was constructed. Furthermore, 109 complete genomes of NDV (seven strains belonged to genotype XX and the other 102 strains belonged to genotype VI) were compared using AliView software, and the datasets containing the coding region sequences (CDS) of six genes of NDVs were constructed separately for selection pressure analysis.

### Phylogenetic Analysis and Demographic History

Based on 427 complete F gene sequences of NDV, a maximum likelihood (ML) tree was constructed using the GTR + F + R4 model as suggested by ModelFinder (37) and was implemented in IQ-TREE with 1,000 bootstrap replicates (38). To verify the temporal structure of all PPMV-1 isolates in China, we regressed root-to-tip genetic distances against sampling date using TreeTime v.0.8.1 (39). To estimate the evolutionary rate and timescale of PPMV-1 in China, a Bayesian Markov Chain Monte Carlo (MCMC) method was conducted using BEAST v.1.10.4 (40) with a relaxed molecular clock and a Bayesian SkyGrid tree prior. The best-fit nucleotide substitution model (HKY + F + G4) was selected using ModelFinder implemented in PhyloSuite (41) based on Bayesian Information Criterion (BIC). At least three independent MCMC analyses were run for 2 × 10^8^ generations and sampling every 20,000 generations. The first 2 × 10^7^ generations were discarded as burn-in. Tracer v.1.7 was used to estimate the effective sampling size (ESS) values to confirm convergence of chains with all values >200 (42). The inferred maximum clade credibility (MCC) tree was produced with TreeAnnotator v.1.10.4 (part of the BEAST package) and visualized in FigTree v.1.4.4 (http://tree.bio.ed.ac.uk/software/figtree/). The SkyGrid reconstruction was implemented to investigate the historical population dynamics of PPMV-1 isolates prevailing in China.

### Discrete Phylogeographic Analysis

To gain insight into the spatial and temporal dynamics of PPMV-1 in China, Bayesian phylogeographic analysis was performed to infer the migration pathways by using BEAST v.1.10.4. Each F gene sequence was assigned two features representing its sampling date and geographical location. China was divided into seven geographical regions: Northeast China (Heilongjiang, Jilin, and Liaoning), North China (Beijing, Tianjin, Hebei, Shanxi, and Inner Mongolia), Central China (Henan, Hubei, and Hunan), East China (Shandong, Jiangsu, Anhui, Shanghai, Zhejiang, Jiangxi, Fujian, and Taiwan), South China (Guangdong, Guangxi, Hainan, Hong Kong, and Macau), Northwest China (Qinghai, Xinjiang, Gansu, Shaanxi, and Ningxia) and Southwest China (Tibet, Sichuan, Chongqing, Guizhou, and Yunnan). The seven regions were chosen and coded as discrete states. The spatial diffusions among different regions were subsequently allowed to be asymmetric and model averaging was carried out by utilizing Bayesian stochastic search variable selection (BSSVS) (43, 44). SpreaD3 v.0.9.7 was used to visualize the spreading process over time and calculate the Bayes factor (BF) support for each route (45). Significant migration routes were determined based on the established criteria (BF ≥ 3 and the posterior probability >0.5). 3 ≤ BF < 10, 10 ≤ BF < 100, 100 ≤ BF < 1,000 and BF ≥ 1,000 indicated substantial, strong, very strong and decisive support, respectively.

### Selective Pressure Analysis

To analyze the positive selection sites in different genes of PPMV-1 isolates prevailing in China, the Fixed Effects Likelihood (FEL) method, the Mixed Effects Model of Evolution (MEME) method, the Fast Unconstrained Bayesian AppRoximation (FUBAR) method and Single Likelihood Ancestor Counting (SLAC) method provided on the Datamonkey website (http://www.datamonkey.org/) were used in this study. Significant levels of *p* < 0.1 in SLAC, *p* < 0.05 in FEL, *p* < 0.05 in MEME and posterior probabilities >0.9 in FUBAR were considered as an indication of positive selection.

### Amino Acid Substitutions Analysis

Previous studies have shown that PPMV-1 is an antigenic variant of chicken-derived NDV (14, 46, 47). To clearly elucidate the alteration of specific amino acid residues in all proteins of PPMV-1 (102 strains belonging to genotype VI) compared with chicken-origin NDV (7 strains belonging to genotype XX), this study used AliView to compare the complete genomes of 109 NDV strains.

### Statistical Analysis

Statistical analysis was carried out using GraphPad Prism version 8.0 (GraphPad Software Inc., San Diego, CA). Error bars in the data from virus growth kinetics indicate standard errors of the means. *P*-values were evaluated by one-way ANOVA to determine the significance level of the difference between distinct experimental groups. Statistical significance was taken as follows: ^*^P ≤ 0.05, significant; ^**^P ≤ 0.01, very significant; ^***^P ≤ 0.001, extremely significant.

## Results

### Isolation and Biological Characterization of PPMV-1 Strains

**Figure 1:**
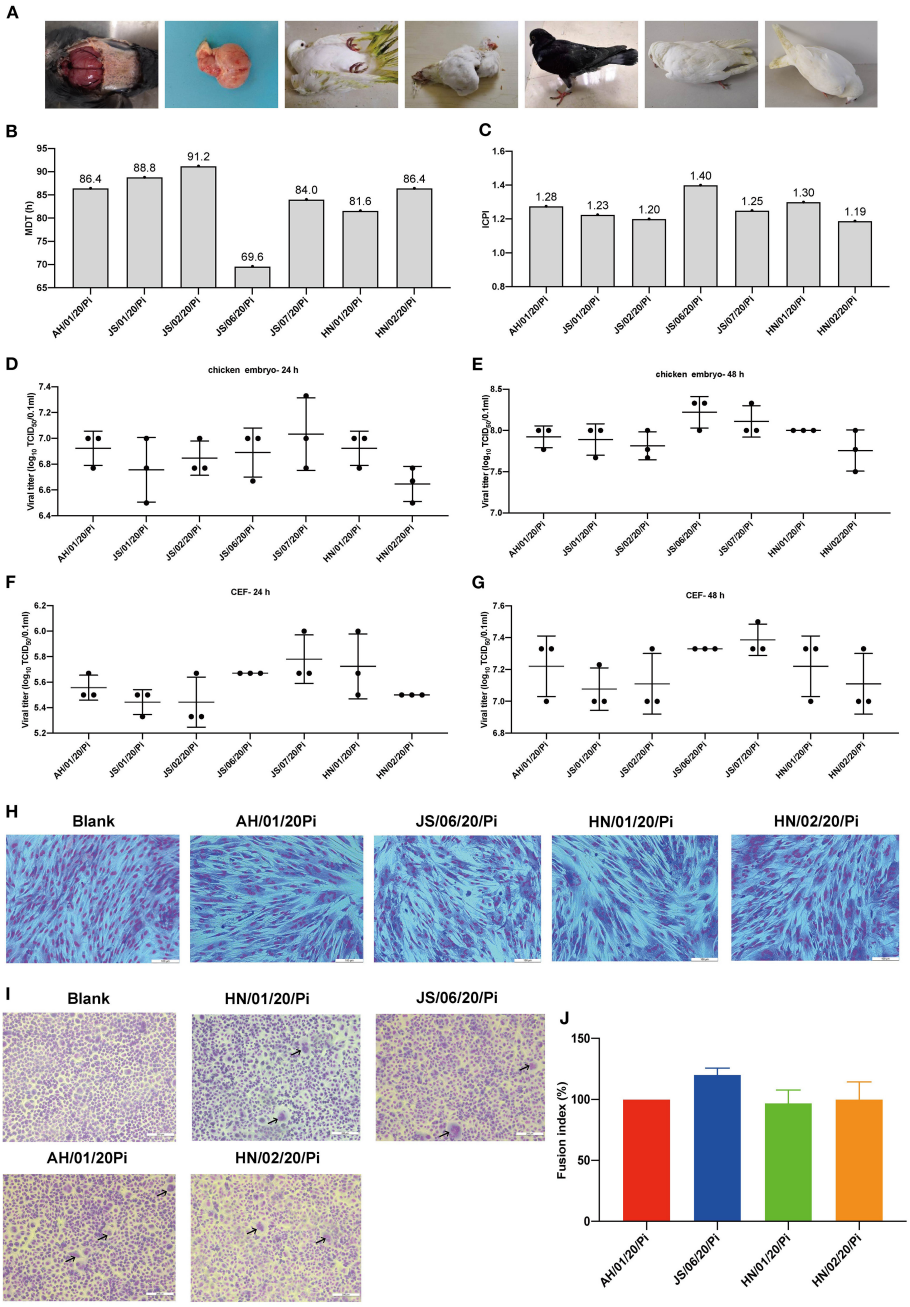
Isolation and biological characteristics of 7 PPMV-1 isolates in this study. (**A)** Clinical signs and gross lesions of diseased pigeons from different provinces. The pathogenic potential of these isolates was determined using the MDT **(B)** and ICPI **(C)** assays. The growth kinetics of different PPMV-1 isolates in 10-day-old SPF chicken embryos at 24 hpi **(D)** and 48 hpi **(E)**. The growth curves of seven PPMV-1 isolates in CEFs at 24 hpi **(F)** and 48 hpi **(G)**. **(H)** CEFs were infected with the indicated MOI of AH/01/20/Pi, JS/06/20/Pi, HN/01/20/Pi, and HN/02/20/Pi at 48 hpi and stained with Giemsa solution to observe CPE. **(I)** Syncytium formation induced by AH/01/20/Pi, JS/06/20/Pi, HN/01/20/Pi, and HN/02/20/Pi. **(J)** Comparison of the fusion index values for AH/01/20/Pi, JS/06/20/Pi, HN/01/20/Pi, and HN/02/20/Pi. Data represent the means ± standard deviations of the results obtained from three independent experiments. Asterisks indicate the significance of the difference. *P*-values were calculated by one-way ANOVA and categorized as *P ≤ 0.05 (significant), **P ≤ 0.01(very significant) and ***P ≤ 0.001 (extremely significant), respectively.

In the present study, 7 PPMV-1 strains were isolated from diseased pigeons from three different provinces (Anhui, Jiangsu, and Henan) during January 2020-December 2020 (Figure 1A and Table 1). The clinical signs of the diseased pigeons contained marked depression, green diarrhea, paralysis, neurological symptom, and the gross lesion included severe congestion in the brain (Figure 1A). The HA titers of the PPMV-1 isolates ranged from 5 log2 to 6 log2 (Table 1). The complete genomes of seven PPMV-1 strains were determined. The amino acid sequences of the F protein cleavage site (^112^RRQKRF^117^) of these isolates were indicative of virulent viruses (Table 1). Nevertheless, the MDT values (Figure 1B) for all isolates were between 69.6 and 91.2 h and the ICPI values (Figure 1C) ranged from 1.19 to 1.40, implying that all isolates were moderately virulent (mesogenic). Additionally, the results showed that all isolates exhibited similar growth kinetics in chicken embryos and CEFs at 24 and 48 hpi (Figures 1D–G). The cytopathic effect assay indicated that the four PPMV-1 isolates (AH/01/20/Pi, JS/06/20/Pi, HN/01/20/Pi, and HN/02/20/Pi) could induce severe CPE including cell rounding, cell shrinkage, cell lysis and formation of large syncytia in CEFs at 48 hpi (Figure 1H). The syncytia formation assays showed that the four isolates (AH/01/20/Pi, JS/06/20/Pi, HN/01/20/Pi, and HN/02/20/Pi) could lead to syncytium formation at 36 hpi (Figure 1I). However, these results suggested that there was no significant difference in the fusion activity of the four isolates (AH/01/20/Pi, JS/06/20/Pi, HN/01/20/Pi, and HN/02/20/Pi) (Figure 1J).

### Phylogenetic Analysis

**Figure 2:**
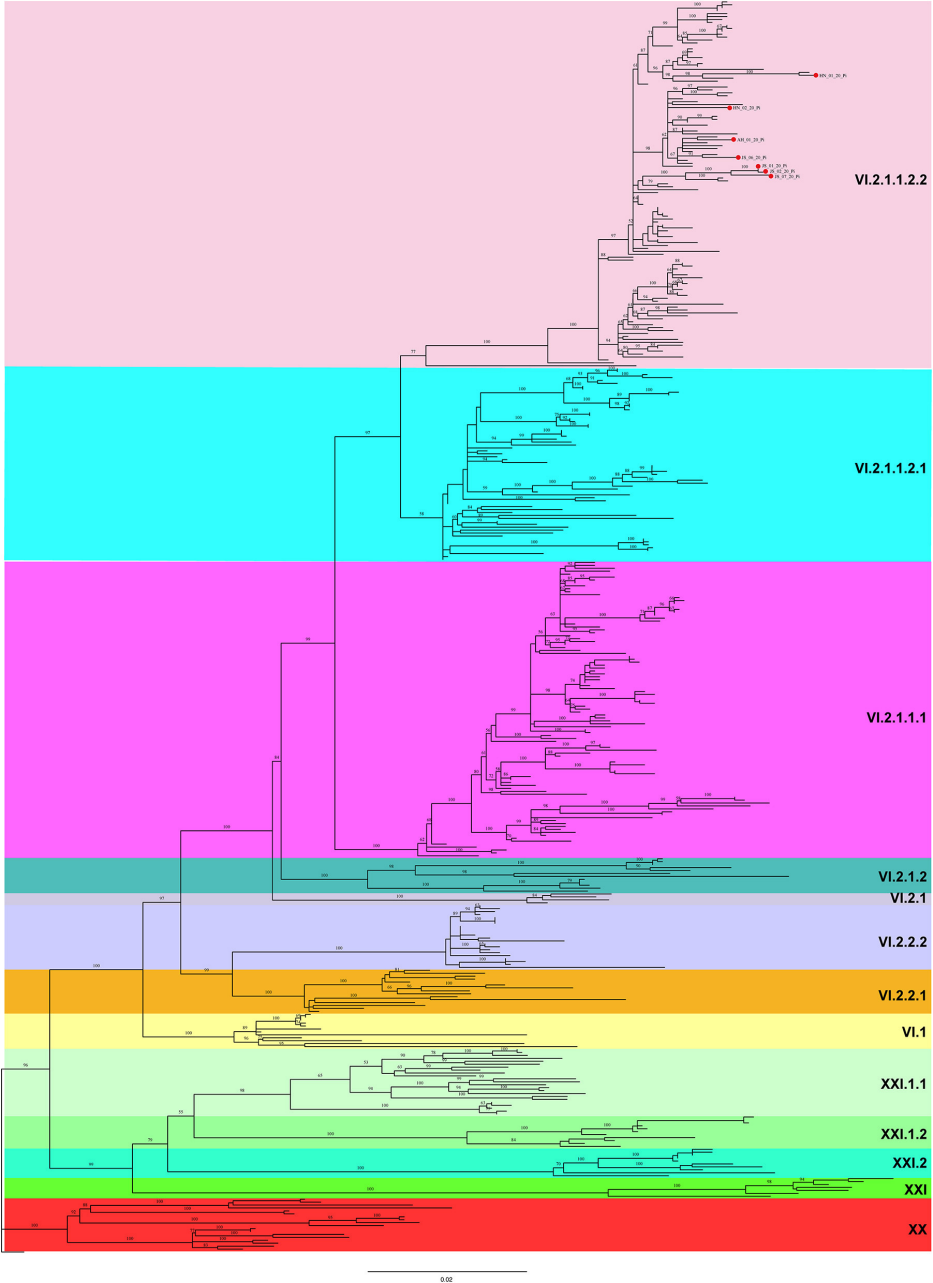
A maximum likelihood (ML) tree based on the complete F gene sequence. The ML tree was constructed using IQ-TREE software with the GTR + F + R4 model (1,000 bootstrap replicates). All 7 PPMV-1 isolates identified in this study are indicated by red solid circles. Different background colors represent different sub-genotypes.

A ML phylogenetic tree was constructed based on the complete F gene sequences. As shown in Figure 2, all 7 PPMV-1 isolates identified in this study were classified as sub-genotype VI.2.1.1.2.2. Besides, the majority of PPMV-1 isolates worldwide belonged to genotype VI, among which 186 PPMV-1 strains were isolated from China. See Supplementary Figure S1 for full details.

**Figure 3:**
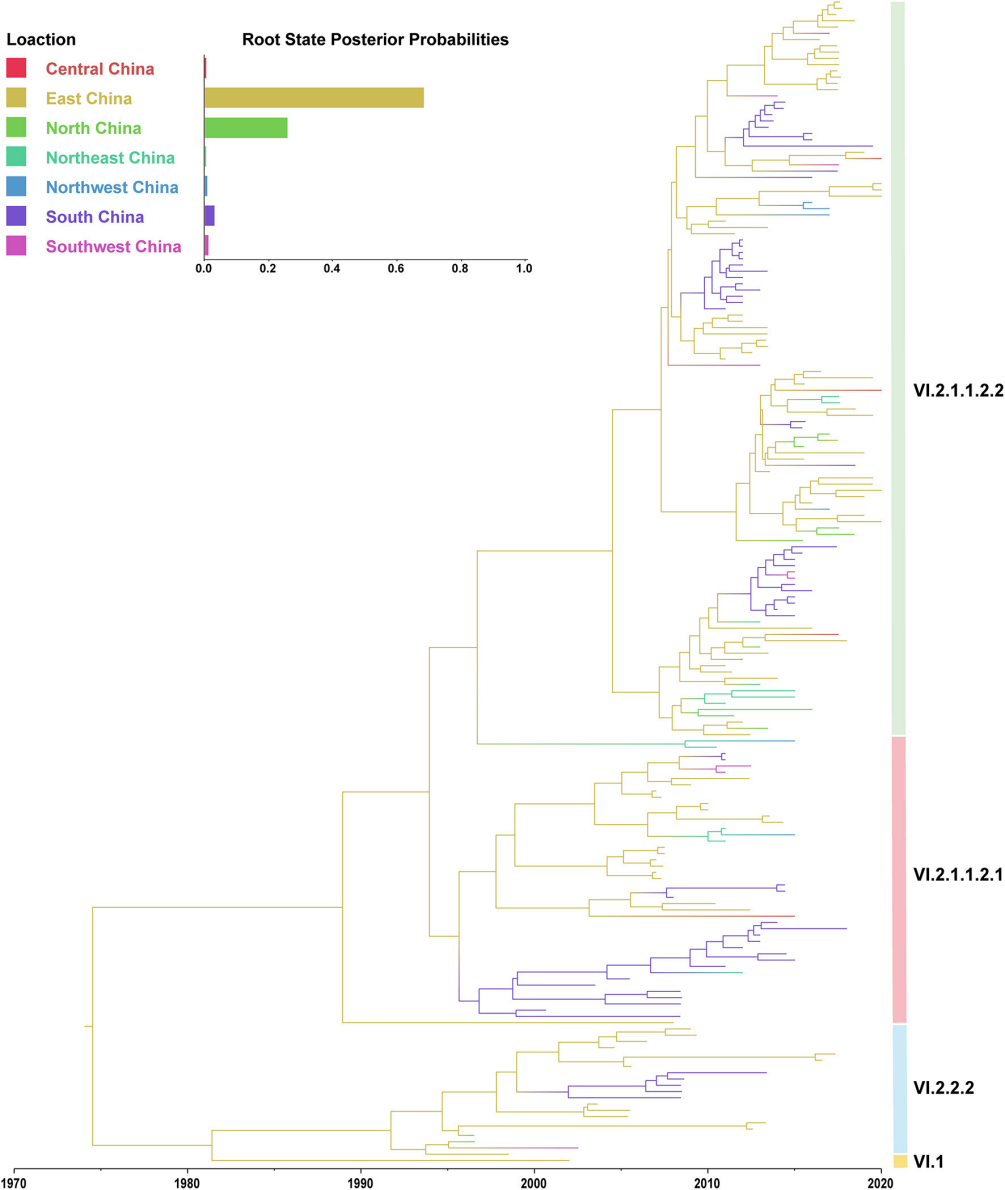
A maximum clade credibility (MCC) tree of the complete F gene of PPMV-1 isolates circulating in China. The MCC tree was constructed using BEAST v.1.10.4. The root state posterior probabilities for seven different regions are exhibited in the embedded panel.

**Figure 4:**
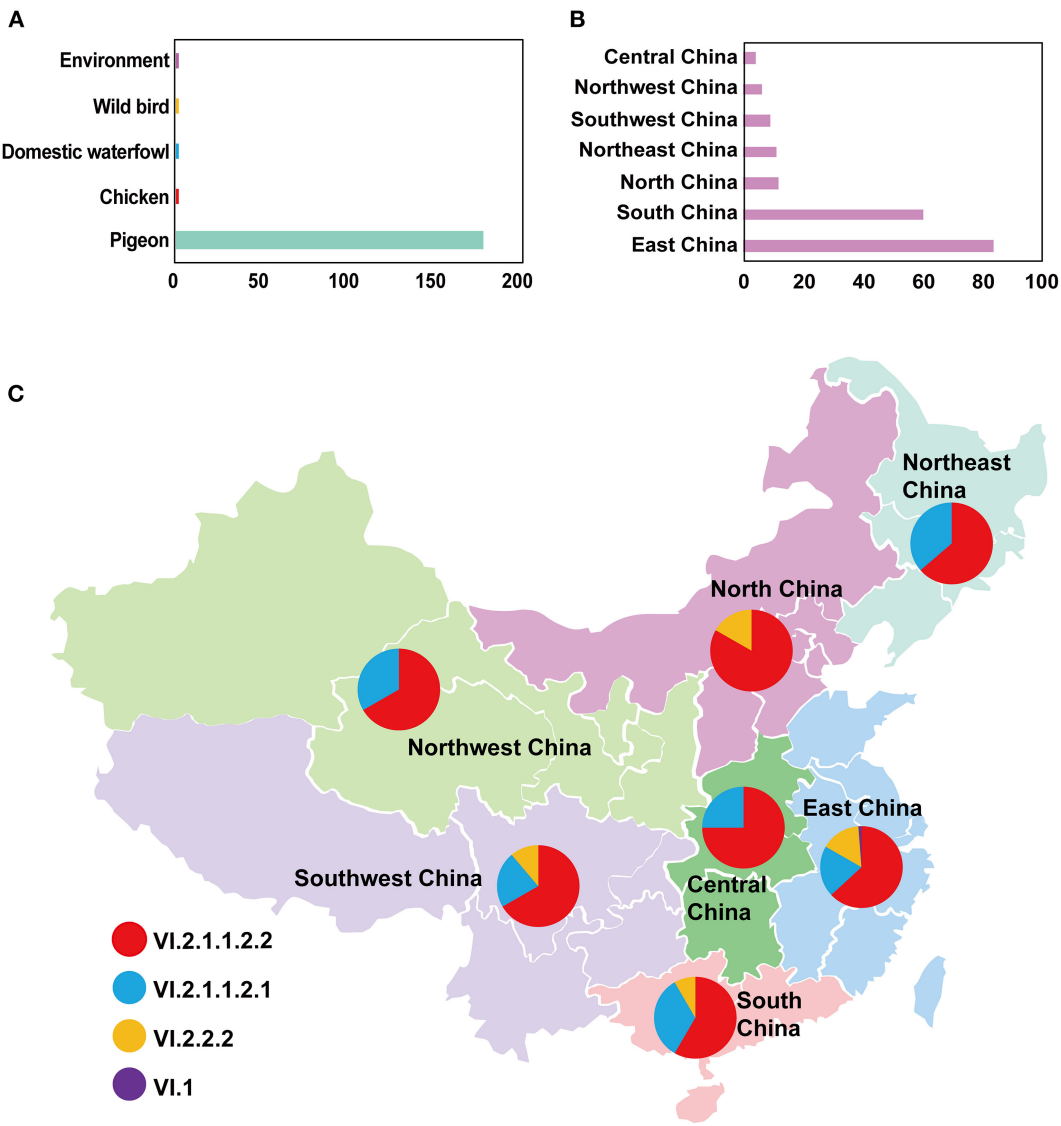
The detailed information of PPMV-1 isolates prevailing in China. **(A)** The host source of all PPMV-1 isolates from China; **(B)** The distribution of PPMV-1 strains isolated from China; **(C)** The proportion of sub-genotypes of PPMV-1 isolates in different regions of China.

In this study, the root-to-tip regression analysis of PPMV-1 strains isolated from China demonstrated the presence of a temporal structure (*R*^2^ = 0.56). The results showed that the evolutionary rate of F gene of PPMV-1 circulating in China which was estimated using Bayesian method was 1.21 × 10^−3^ subs/site/year (95% confidence interval, 9.76 × 10^−4^-1.47 × 10^−3^). The time-scaled MCC tree of PPMV-1 in China based on the complete F gene suggested that all 186 viruses belonged to four different sub-genotypes, with sub-genotype VI.2.1.1.2.2 (118/186, 63.4%) being the most abundant, followed by sub-genotype VI.2.1.1.2.1 (46/186, 24.7%), sub-genotype VI.2.2.2 (21/186, 11.3%), and sub-genotype VI.1 (1/186, 0.5%) (Figure 3). The Bayesian analysis also indicated that all PPMV-1 isolates were rooted with the virus strains from East China, and PPMV-1 might first emerge in East China (posterior probability = 0.69), with the time of most recent common ancestor (TMRCA) around 1974 (95% highest posterior density (HPD), 1956–1986) (Figure 3). See also Supplementary Figure S2 for details. The statistics of the host sources revealed that most of PPMV-1 strains (178/186) were isolated from pigeons, which further indicated that PPMV-1 has a high host preference for pigeons (Figure 4A). In terms of the spatial distribution of the 186 PPMV-1 strains (Figure 4B), the largest number of viruses came from East China (84/186, 45.2%), followed by South China (60/186, 32.3%), North China (12/186, 6.5%), Northeast China (11/186, 5.9%), Southwest China (9/186, 4.8%), Northwest China (6/186, 3.2%), and Central China (4/186, 2.2%). It is noteworthy that sub-genotype VI.2.1.1.2.2 accounted for the largest proportion of viruses in all seven regions of China, and East China had the greatest diversity of sub-genotypes (VI.1, VI.2.2.2.2, VI.2.1.1.2.1, and VI.2.1.1.2.2) (Figure 4C).

### Demographic History

**Figure 5:**
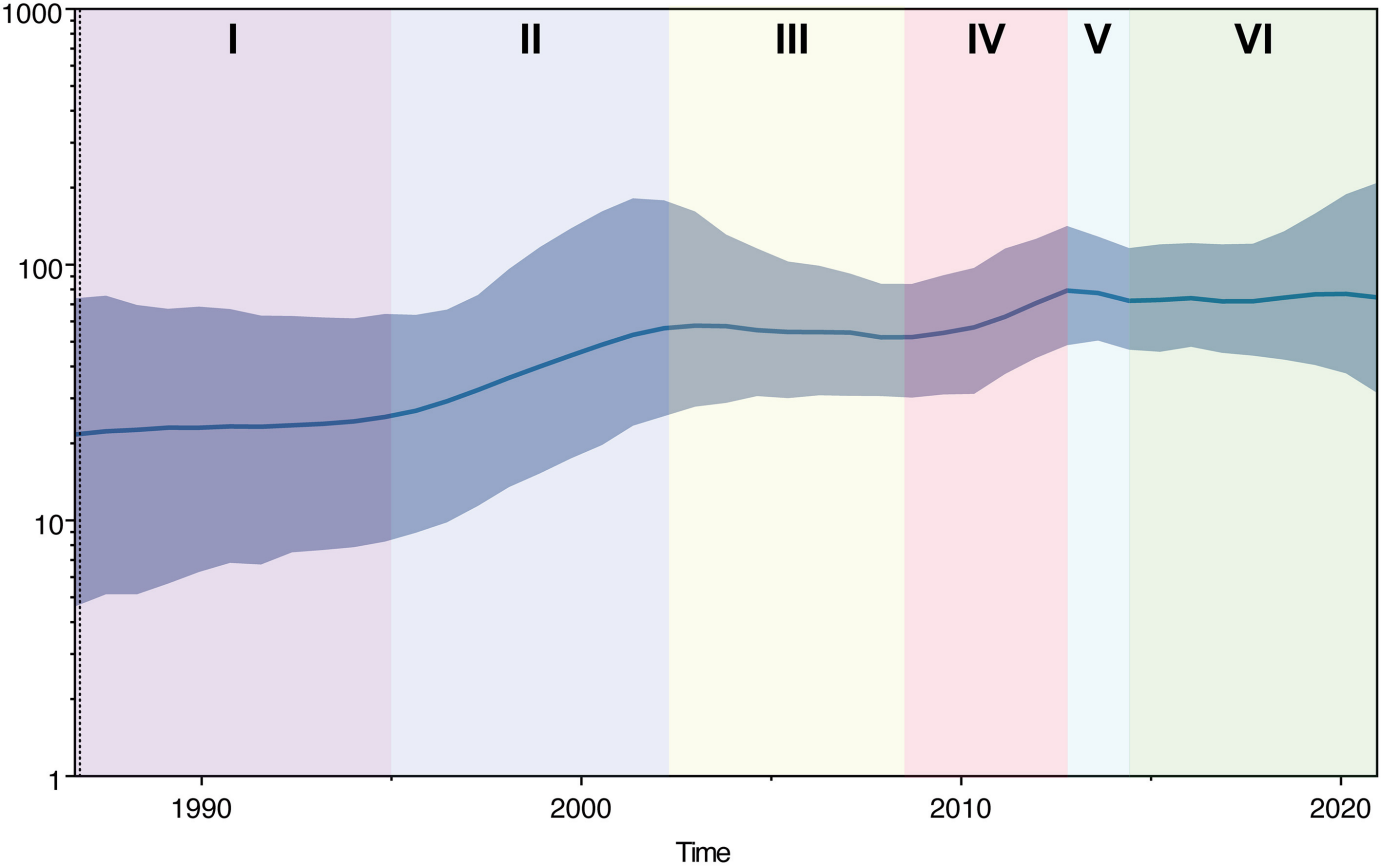
Bayesian SkyGrid plot displaying the changes in effective population size of PPMV-1 strains circulating in China over time. The dark blue line denotes the mean value of genetic diversity and the light blue shading indicates the 95% confidence interval. Different colors represent different phases.

The historical population dynamics of PPMV-1 circulating in China indicated that the change in the population size might go through six stages (I-VI) (Figure 5). In the first stage (before 1995), the population size of PPMV-1 isolates in China remained relatively constant. Then, the PPMV-1 population expanded at stage II (1995–2002). In the third stage (2003–2008), the population size of PPMV-1 remained basically constant. Next, the PPMV-1 population increased rapidly again at stage IV (2009–2013). Nevertheless, the population size of PPMV-1 decreased slightly at stage V (2013–2014). Subsequently, the PPMV-1 population reached a relatively constant level at stage VI (after 2014).

### Migration Pattern of PPMV-1 in China

**Figure 6:**
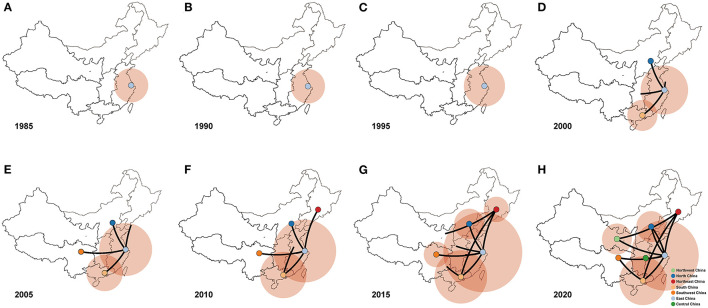
Spatiotemporal dynamics of PPMV-1 isolates among different regions in China represented with snapshots of the dispersal pattern for **(A)** 1985, **(B)** 1990, **(C)** 1995, **(D)** 2000, **(E)** 2005, **(F)** 2010, **(G)** 2015, and **(H)** 2020.

We conducted Bayesian phylogeographic analysis to elucidate the spatial transmission patterns of PPMV-1 in seven geographic regions of China (East China, South China, North China, Northeast China, Southwest China, Northwest China, and Central China). The results showed that the presence of PPMV-1 might limit to East China before 1995 (Figures 6A–C), and then spread from East China to North China, South China, and Southwest China from 2000 to 2005 (Figures 6D,E). PPMV-1 started to spread to other regions from South China by the year 2010 (Figure 6F). Since then, South China has become another new epicenter besides East China (Figure 6G). Notably, PPMV-1 has been found in all seven geographic regions of China by 2020 (Figure 6H).

**Figure 7:**
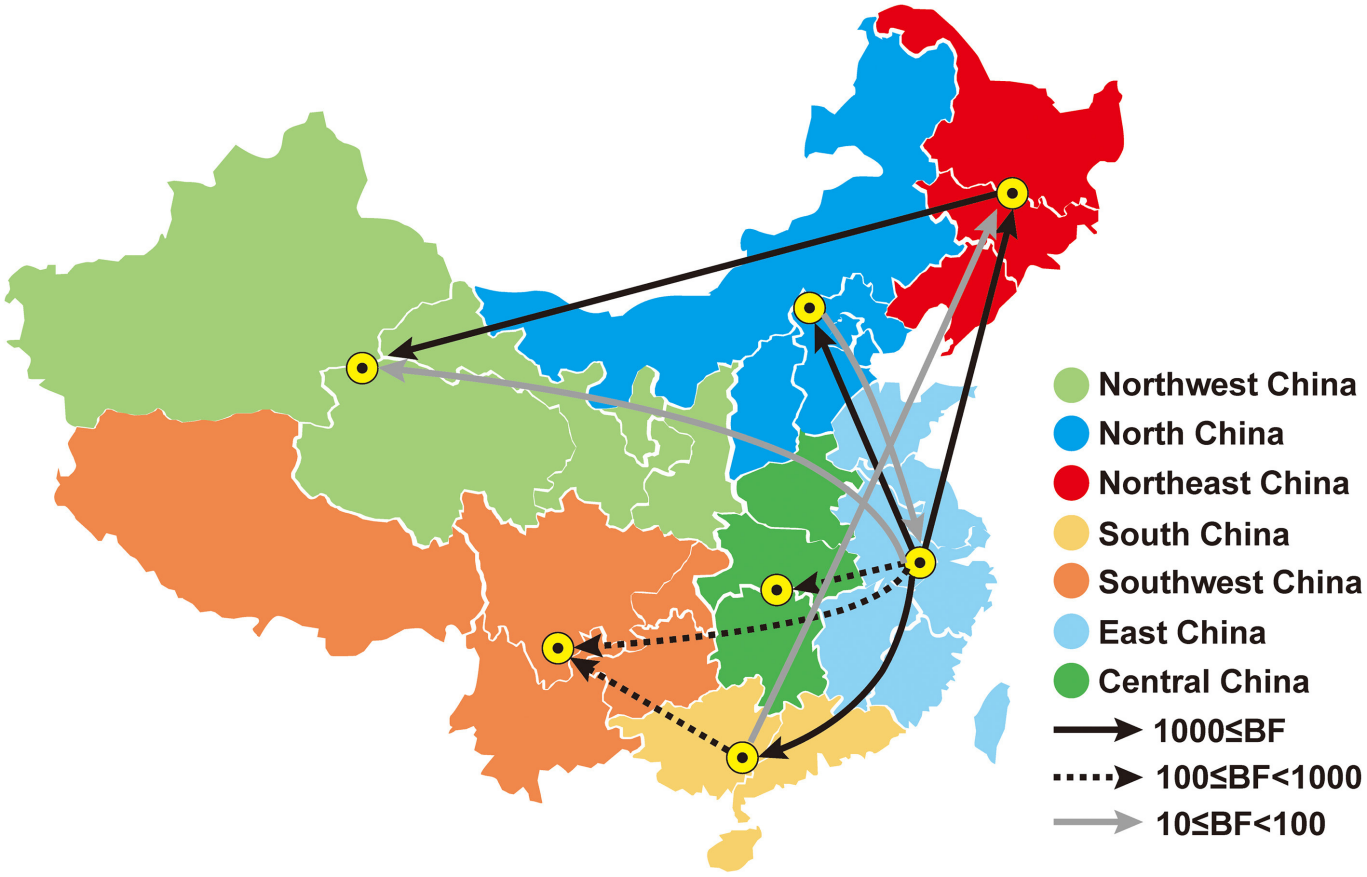
Spatial diffusion of PPMV-1 isolates in China. Statistically supported migrations with Bayes factor (BF) > 3 are presented. Morphological characteristics of lines represents migration rates: black solid arrows, decisive support with BF ≥ 1,000; black dashed arrows, very strong support with 100 ≤ BF < 1,000; gray solid arrows, strong support with 10 ≤ BF < 100.

As exhibited in Figure 7 and Table 3, these outcomes demonstrated that there were 10 migration links in the diffusion of PPMV-1 in China. The highest average transition rate was observed for migration from East China to South China, while the lowest mean transition rate was found for migration from East China to Northwest China (Table 3). Moreover, East and South China might be the two main epicenters responsible for eight migration routes of PPMV-1 (Figure 7). Among the eight migration routes, three transmission routes are decisively supported, including migration from East to South China (BF = 47475.674), from East to North China (BF = 47475.674), and from East to Northeast China (BF = 3647.106). The routes from East China to Central China (BF = 274.025), from East China to Southwest China (BF = 159.589) and from South China to Southwest China (BF = 161.325) had very strong support. The routes from East to Northwest China (BF = 85.511) and from South China to Northeast China (BF = 33.804) had strong support. Additionally, the route from Northeast China to Northwest China had decisive support (BF = 1577.423). The route from North China to East China also had strong support (BF = 11.731). Overall, these above results suggested that East China and South China might play a critical role in seeding PPMV-1 throughout China.

**Table 3:** Statistically supported migration rates of PPMV-1 isolates circulating in China estimated from the F gene.

**From**	**To**	**Mean transition rate**	**Bayes factor^a^**	**Posterior probability^b^**
East China	North China	1.799	47475.674	1.000
East China	Northeast China	1.158	3647.106	0.999
East China	Northwest China	0.668	85.511	0.942
East China	South China	2.069	47475.674	1.000
East China	Southwest China	1.022	159.589	0.968
East China	Central China	0.807	274.025	0.981
South China	Southwest China	0.813	161.325	0.968
South China	Northeast China	0.809	33.804	0.865
Northeast China	Northwest China	1.379	1577.423	0.997
North China	East China	1.631	11.731	0.690

a *Statistically supported transitions with BF > 3 are presented*.

b *Posterior Probability > 0.5 suggests well-support viral transition*.

### Positive Selection Sites in Different Genes of PPMV-1 in China

To gain a better understanding of the selection pressure acting on different genes of PPMV-1 in China, we conducted a positive selection analysis using four disparate detection methods (FEL, MEME, FUBAR, and SLAC methods). As shown in Table 4, three codons (sites 8, 432 and 486) were found to be under positive selection in the NP gene of PPMV-1 according to one detection method. Furthermore, three positive selection sites (47, 89, and 192) were simultaneously predicted in the P gene by the four methods. One positive selection site (73) was detected in the M gene by the FUBAR method. Within F gene, two positive selection sites (4 and 28) were detected based on all four methods, whereas one positive selection site (13) was predicted using two methods. In addition, four positive selection sites (92, 115, 266, and 407) were identified in the HN gene by using one method. One positive selection site (1,672) was identified by all four methods, and the other positive selection site (1,365) in the L gene was detected by both MEME and FUBAR methods, Moreover, six positive selection sites (147, 1,759, 1,804, 2,013, 2,029, and 2,064) were found in the L gene of PPMV-1 by one method. Combining the above results, this study demonstrated that the majority of the positive selection sites were located at the NP, P and L genes.

**Table 4:** The selection profile of different genes of PPMV-1 in China.

**Gene**	**FEL^a^**	**MEME^b^**	**FUBAR^c^**	**SLAC^d^**
NP	/^e^	8, 486	432	/
P	47^*^^f^, 89*, 168*, 192*	47*, 73, 89*, 100, 168*, 169,192*	47*, 49, 89*, 168*, 192*, 319	47*, 89*, 192*
M	/	/	73	/
F	4*, 13*, 28*	4*, 6, 28*, 139, 474, 553	4*, 28*	4*, 13*, 18, 28*
HN	/	92, 115, 407	266	/
L	1,672*	1,365*, 1,672*, 1,759, 1,804, 2,013, 2,029, 2,064	147, 1,365*, 1,672*	1,672*

a *Fixed Effects Likelihood*.

b *Mixed Effects Model of Evolution*.

c *Fast Unconstrained Bayesian AppRoximation*.

d *Single Likelihood Ancestor Counting*.

e *A slash (/) indicates that no positive selection site was found by the corresponding method*.

f An asterisk (*) represents a site which was simultaneously predicted as a positive selection site by two or more methods.

### Amino Acid Substitutions Analysis

It has been shown that PPMV-1 might be an antigenic variant of chicken-origin NDV (14, 46, 47). To investigate the amino acid differences between PPMV-1 and chicken-origin NDV, we compared 109 full-length sequences in this study. The results presented in Table 5 showed the presence of specific amino acid substitutions in all six proteins of PPMV-1 compared to chicken-origin NDV: six different substitutions (G106S, A154V, S405N/K, N463D, T469I, and P477H) in the NP protein; seven disparate substitutions (D10E, S92P, N/I93K/R, E103D/N/G, R163G, A343V/M, and R380K) in the P protein; two distinct substitutions (Q36R and M77T) in the M protein; one different substitution (V50I) in the F protein; six distinct substitutions (V9A, Y112H, G304S, E/K347G, D349E, and A540V/I) in the HN protein; ten disparate substitutions (T129A, T340S, M1079T/I, V1082I, S1099P, N1110A/T, F/S1727P/L, A1757V, K2120R, and D/N2173E/G) in L protein (Table 5). In summary, PPMV-1 had 23 specific amino acid substitutions in the NP, P, and L proteins and nine specific amino acid substitutions in the M, F and HN proteins compared with chicken-origin NDV.

**Table 5:** The special amino acid substitutions in different proteins of PPMV-1 compared with chicken-origin NDV.

**Gene**	**Amino acid position**	**Chicken-origin NDV^a^**			**PPMV-1^b^**		
NP	106	G (7)^c^			S (102)		
	154	A (7)			V (102)		
	405	S (7)			N (101)		K (1)
	463	N (7)			D (102)		
	469	T (7)			I (102)		
	477	P (7)			H (102)		
P	10	D (7)			E (102)		
	92	S (7)			P (102)		
	93	N (6)		I (1)	K (94)		R (8)
	103	E (7)		D (92)	N (9)		G (1)
	163	R (7)			G (102)		
	343	A (7)			V (101)		M (1)
	380	R (7)			K (102)		
M	36	Q (7)			R (102)		
	77	M (7)			T (102)		
F	50	V (7)			I (102)		
HN	9	V (7)			A (102)		
	112	Y (7)			H (102)		
	304	G (7)			S (102)		
	347	E (6)		K (1)	G (102)		
	349	D (7)			E (102)		
	540	A (7)			V (101)		I (1)
L	129	T (7)			A (102)		
	340	T (7)			S (102)		
	1,079	M (7)			T (101)		I (1)
	1,082	V (7)			I (102)		
	1,099	S (7)			P (102)		
	1,110	N (7)			A (93)		T (9)
	1,727	F (6)		S (1)	P (93)		L (9)
	1,757	A (7)			V (102)		
	2,120	K (7)			R (102)		
	2,173	D (6)		N (1)	E (101)		G (1)

a *7 strains belonging to genotype XX*.

b *102 strains belonging to genotype VI*.

c *Values in parentheses indicate the number of occurrences of the amino acid*.

## Discussion

Since the introduction of PPMV-1 from Europe into China, it has long been prevalent among pigeon flocks for a long time, leading to significant economic losses in the pigeon industry nationwide (23, 25, 26, 48). In this study, we identified seven PPMV-1 strains from diseased pigeons in Jiangsu, Anhui, and Henan provinces during 2020. Although the F protein cleavage site sequences of seven PPMV-1 strains were typical of velogenic viruses, these PPMV-1 isolates belonged to mesogenic strains based on MDT and ICPI values which indicated that other structural domains or genes might influence viral virulence in addition to the F protein cleavage site (31, 49).

Phylogenetic analysis showed that these seven viruses were classified as sub-genotype VI.2.1.1.2.2. Additionally, all PPMV-1 isolates in China were clustered into four sub-genotypes (VI.2.1.1.2.2, VI.2.1.1.2.1, VI.2.2.2, and VI.1). Notably, sub-genotype VI.2.1.1.2.2 accounted for the majority of the PPMV-1 strains which indicated that sub-genotype VI.2.1.1.2.2 has become the dominant sub-genotype in China (26, 27). From the host distribution, most of PPMV-1 strains (178/186) were isolated from pigeons, further confirming that PPMV-1 has a significant host preference for pigeons (15). The PPMV-1 strains isolated from East China were located at the root of the MCC tree with the highest posterior probability of 0.69, suggesting that East China was probably the origin of PPMV-1 in China. Moreover, TMRCA of PPMV-1 isolates was dated to the year 1974 (95% HPD: 1956–1986), well before the first genome sequence of the PPMV-1 strain from China was submitted to GenBank. Notably, sample bias, scant sampling and sequencing might potentially influence this conclusion, especially given the scarcity of data in the epidemiological surveillance in the last century (27, 50). Over the last 25 years, these viruses transmitted from East China to the rest of the nation and East China and South China were likely the major epicenters responsible for the dissemination of PPMV-1. The suitable climatic conditions, convenient transportation and high population density could account for that East China and South China might play a critical role in the spread of PPMV-1. Hence, strengthening the monitoring of PPMV-1 in East and South China can contribute to understanding the evolutionary dynamics of PPMV-1 in these two regions and predicting the epidemic in other regions in a timely manner, as well as providing a theoretical basis for the development of reasonable PPMV-1 prevention and control strategies.

The demographic history indicated that the population size change of PPMV-1 in China might have gone through six different phases. The population size of PPMV-1 in China remained relatively constant during phase I (prior to 1995), which might be attributed to the low demand for the young pigeons and the slow development of the pigeon industry during this period. The population of PPMV-1 increased rapidly at stage II (1995–2002), probably due to the rapid growth of the pigeon industry in China along with poor feeding management and the lack of a sanitary and epidemic prevention system (50). Then, the PPMV-1 population remained basically constant in the third phase (2003–2008), which was likely caused by the fact that the SARS outbreak dealt a huge blow to the pigeon industry in China during 2002–2003, as well as the increasing use of conventional ND vaccines such as La Sota. The rapid increase in PPMV-1 population size at phase IV (2009–2013) could be because of the following two reasons: on the one hand, the pigeon industry entered a new period of rapid expansion with the increasing demand of the young pigeons in China; on the other hand, in the scenario where the circulating PPMV-1 isolates (genotype VI) were sufficiently divergent from the La Sota vaccine strain (genotype II), the vaccine-induced immune response did not confer better protection and prevent virus shedding (51). Interestingly, the population size of PPMV-1 in China decreased slightly at stage V (2013–2014), likely because of the significant impact of H7N9 avian influenza epidemic on the pigeon industry within this period. Subsequently, the PPMV-1 population remained relatively constant at stage VI (after 2014) which might be explained by the fact that although the pigeon breeding industry was able to gradually recover and entered a new stage of development as the demand for suckling pigeons increased, intensive farms with better biosafety developed rapidly with the decrease of individual backyard farms.

Adaptive evolution of genes and genomes determines evolutionary morphology, innovation and species divergence (52, 53). Previous studies have shown that PPMV-1 might be a variant of chicken-derived NDV (14, 46, 47). Besides, our previous study indicated that PPMV-1 changed in virulence and obtained a survival advantage over chicken- origin NDV in pigeons after undergoing adaptive evolution (31). In this study, selection pressure analysis showed that positive selection sites existed in all six genes of PPMV-1, with most of them located at the NP, P, and L genes. In addition, amino acid substitutions analysis indicated that the majority of specific amino acid substitutions (23/32, 71.9%) were situated in the NP, P, and L proteins of PPMV-1 compared to chicken-origin NDV. The aforementioned findings suggested that the viral replication complex (NP, P, and L proteins) might be associated with the host preference of PPMV-1. However, further validation studies utilizing reverse genetics techniques are needed in the future.

All in all, we isolated seven PPMV-1 strains from Jiangsu, Anhui, and Henan provinces in 2020. This study systematically revealed the diffusion pattern of PPMV-1 isolates among different regions of China. Our findings demonstrated that East China and South China might play a critical role in the dissemination of PPMV-1. This work will be of benefit to us for a better understanding of the evolutionary dynamics of PPMV-1 in China and will have major significance for PPMV-1 prevention and control in China, as well as providing important clues for further in-depth studies on the molecular mechanism of host preference of PPMV-1.

## Data Availability Statement

The datasets presented in this study can be found in online repositories. The names of the repository/repositories and accession number(s) can be found in the article/Supplementary Material.

## Ethics Statement

The animal study was reviewed and approved by the Jiangsu Administrative Committee for Laboratory Animals.

## Author Contributions

XFL, TZ, and DH contributed to the study design. TZ, DH, XLL, TL, WW, and QC conducted the experiments. TZ and DH analyzed the data. TZ wrote the manuscript. MG, XW, and XWL reviewed the study procedures. XFL and SH revised the manuscript. All authors reviewed and approved the final manuscript for submission.

## Funding

This work was sponsored by the National Key Technology R&D Program of China (2015BAD12B03), the Earmarked Fund for China Agriculture Research System (CARS-40), the National Key Research and Development Program of China (2017YFD500101), and a project funded by the Priority Academic Program Development of Jiangsu Higher Education Institutions. This research was supported by the High-Performance Computing Cluster of College of Veterinary Medicine, Yangzhou University.

## Conflict of Interest

The authors declare that the research was conducted in the absence of any commercial or financial relationships that could be construed as a potential conflict of interest.

## Publisher's Note

All claims expressed in this article are solely those of the authors and do not necessarily represent those of their affiliated organizations, or those of the publisher, the editors and the reviewers. Any product that may be evaluated in this article, or claim that may be made by its manufacturer, is not guaranteed or endorsed by the publisher.

## Supplementary Material

The dataset and figures supporting this article have been uploaded as part of the supplementary material.

Supplementary Figure S1 A maximum likelihood (ML) tree showing taxa names based on the complete F gene sequence.

Supplementary Figure S2 A maximum clade credibility (MCC) tree showing taxa names based on BEAST analysis.

Supplementary Dataset S1 The alignment sequence data of NDV used for phylogenetic analysis (Figure 2 and Supplementary Figure S1).
