# Supplementary figures and images for: Biological Characterization and Evolutionary Dynamics of Pigeon Paramyxovirus Type 1 in China
Source: Front Vet Sci. 2021 Oct 13;8:721102. doi: 10.3389/fvets.2021.721102 (PMC8548471; doi:10.3389/fvets.2021.721102)

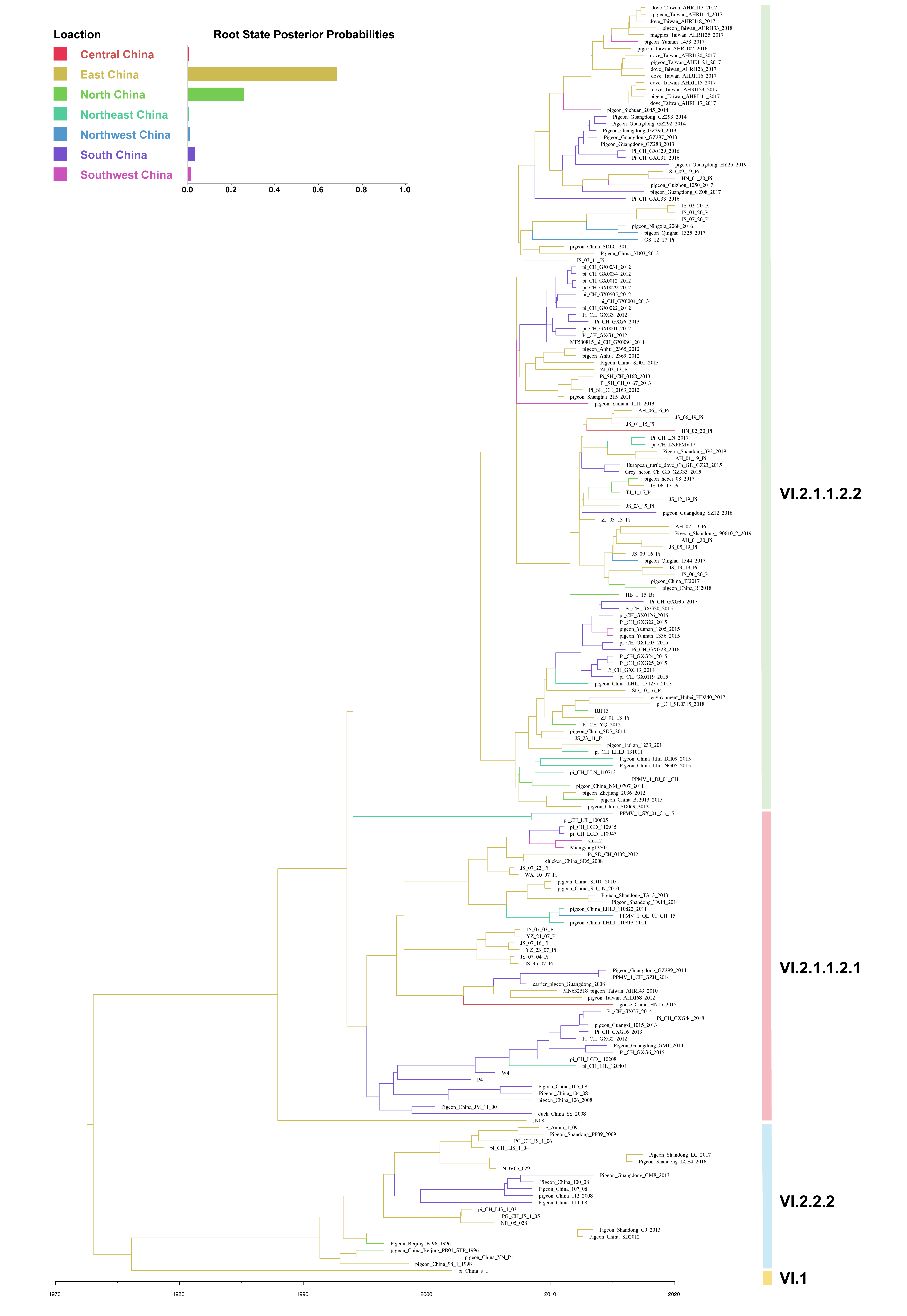

Supplement: Supplementary Figure S2 — A maximum clade credibility (MCC) tree showing taxa names based on BEAST analysis. [file Image_2.TIF]
